# Supplementary material for: Neural signatures of temporal anticipation in human cortex represent event probability density
Source: Nat Commun. 2025 Mar 16;16:2602. doi: 10.1038/s41467-025-57813-7 (PMC11911442; doi:10.1038/s41467-025-57813-7)
Supplement: Supplementary file 1 — Supplementary Information [file 41467_2025_57813_MOESM1_ESM.pdf]

Supplementary Materials for

Neural signatures of temporal anticipation in human cortex represent event probability density

Matthias Grabenhorst*, David Poeppel, Georgios Michalareas
*Corresponding author. Email: m.g@ae.mpg.de

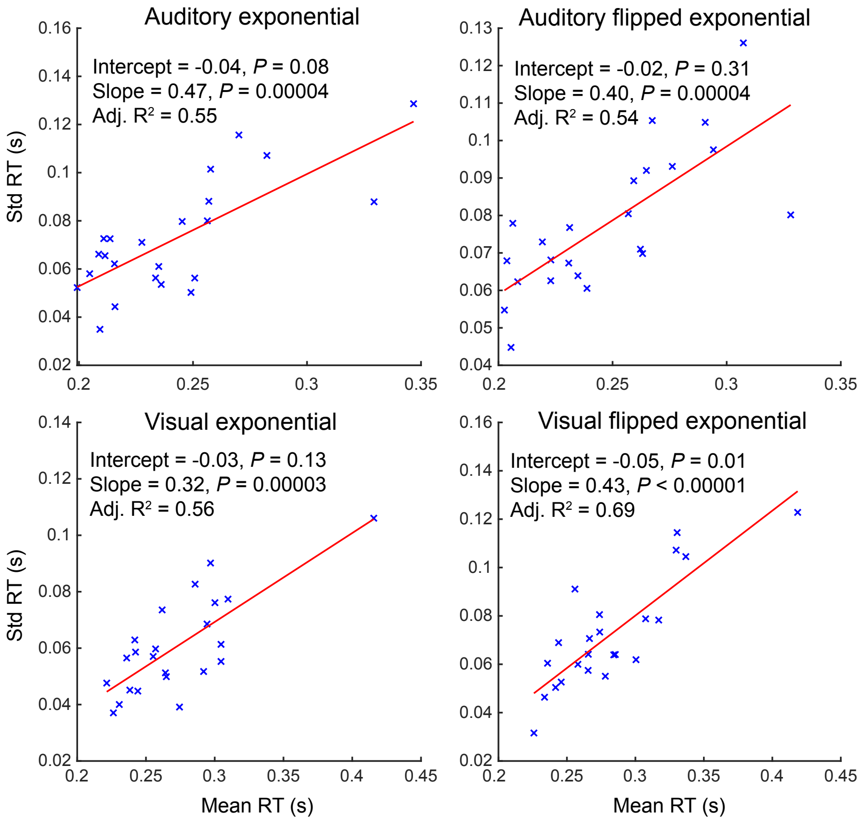

Supplementary Fig 1. | Correlation between mean RT and standard deviation of RT. Within each condition, the standard deviation is positively correlated with mean RT. Both variables were computed within-participant, within-condition and averaged across all trials. Each data point corresponds to a single participant (N = 23). Source data are provided as a Source Data file.

Supplementary Table 1 Number of early response trials (ERT) in which the button was pressed before Go cue, within-condition summed up across all 23 participants.

Modality	Distribution	N all trials	N_{ERT} (% all trials)	mean N_{ERT}
Audition	Exponential	8049	141 (1.71 %)	6.13
Audition	Flipped exponential	8077	119 (1.44 %)	5.17
Vision	Exponential	7946	143 (1.73 %)	6.22
Vision	Flipped exponential	8012	112 (1.35 %)	4.87

Mean N_{ERT} is the average number of ERTs per participant.

Supplementary Table 2 Number of early response trials (ERT) does not differ across conditions.

Contrast	Δ mean N_{ERT}	$t_{(22)}$	*P*
Audition: exp vs. flip exp	0.96	1.20	0.24
Vision: exp vs. flip exp	1.35	1.57	0.13
Exponential: aud vs. vis	-0.087	-0.09	0.93
Flipped exponential: aud vs. vis	0.30	0.26	0.80

Within-condition, number of trials with early response (button pressed before Go cue) was computed per participant. Paired, two-sided t-tests on group-level means across Go time distribution conditions (within modality) and across modality (within Go time distribution conditions).

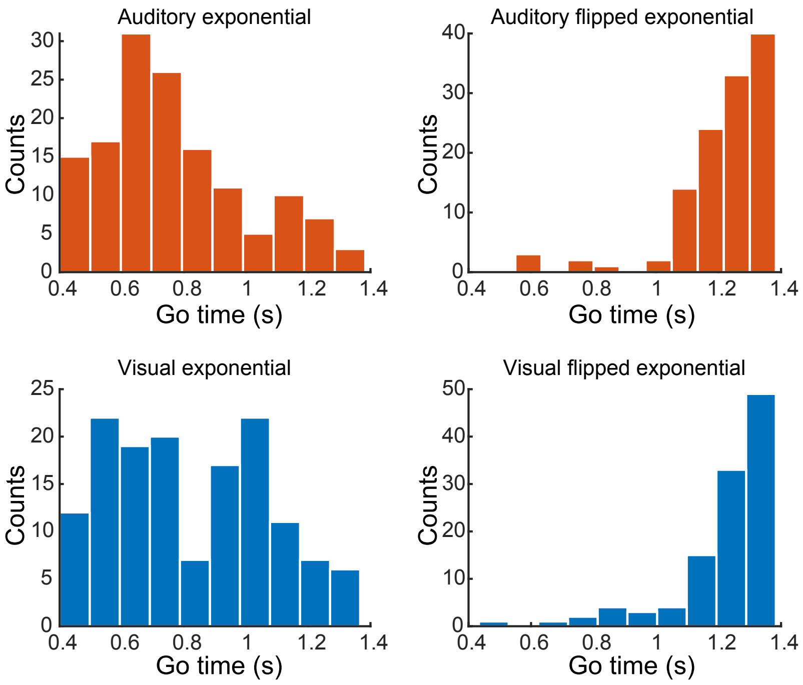

Supplementary Fig. 2 | Number of early response trials over go time

Number of trials with early responses (button pressed before Go cue) pooled across all participants (N = 23) per experimental condition. Early responses were rare (Supplementary Table 1). Participants tended to respond early where there is a large probability of Go cue occurrence, i.e. at shorter go times in the exponential conditions and at longer go times in the flipped exponential conditions. This pattern was more pronounced in the flipped exponential conditions.

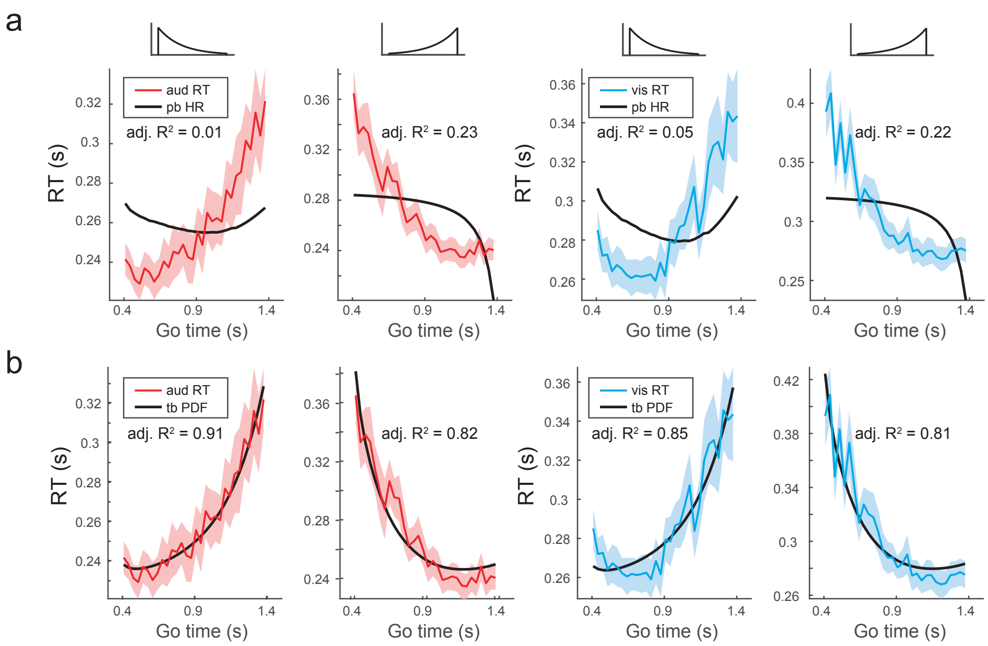

Supplementary Fig 3. | Models based on probabilistically blurred HR and temporally blurred PDF.

a. Fits of mirrored probabilistically blurred HR (Methods) do not capture mean auditory or visual RT. **b**. Reciprocal temporally blurred PDF (Methods) fits mean auditory and visual RT better than the HR model. However, in the flipped exponential conditions, at Go times larger than t = ~1s, the temporally blurred model over-estimates RT in both audition and vision. Note that the model based on the *probabilistically* blurred reciprocal PDF (Fig. 2d and e) outperforms the *temporally* blurred PDF model. **a & b:** For plotting, RTs and fit curves were smoothed by reducing the Go time step size from 16 to 32 ms. Shaded areas represent s.e.m. across participants. Source data are provided as a Source Data file.

Supplementary analysis: Analysis of local minima in negative correlation cluster.

This analysis investigates whether the frequencies at which Spearman's rho reaches a minimum in the alpha and low beta frequency ranges are harmonics of each other. First, the negative cluster was split according to alpha (7 to 12 Hz) and low beta (15 to 22 Hz) frequency ranges, each comprising a set of MEG sensors. Sensors shared by alpha and low beta frequency bands were removed. Within the resultant exclusive sets of alpha and low beta channels, the frequency was identified at which Spearman's rho (averaged across channels and time t = [-0.4, 0] s) reached a minimum. In the alpha range, the minimum is located at f = 7.9 Hz. The theoretical first harmonic of the f = 7.9 Hz is f = 15.8 Hz. In the in the low beta range the minimum is at f = 17.0 Hz. There is a difference of f = 1.2 Hz (17.0 Hz - 15.8 Hz) between the theoretical first harmonic of the alpha minimum and the observed low beta minimum. Therefore, the local minimum in the low beta range is not a harmonic of the local minimum of the alpha range, supporting the separation into alpha and low beta fequency bands.

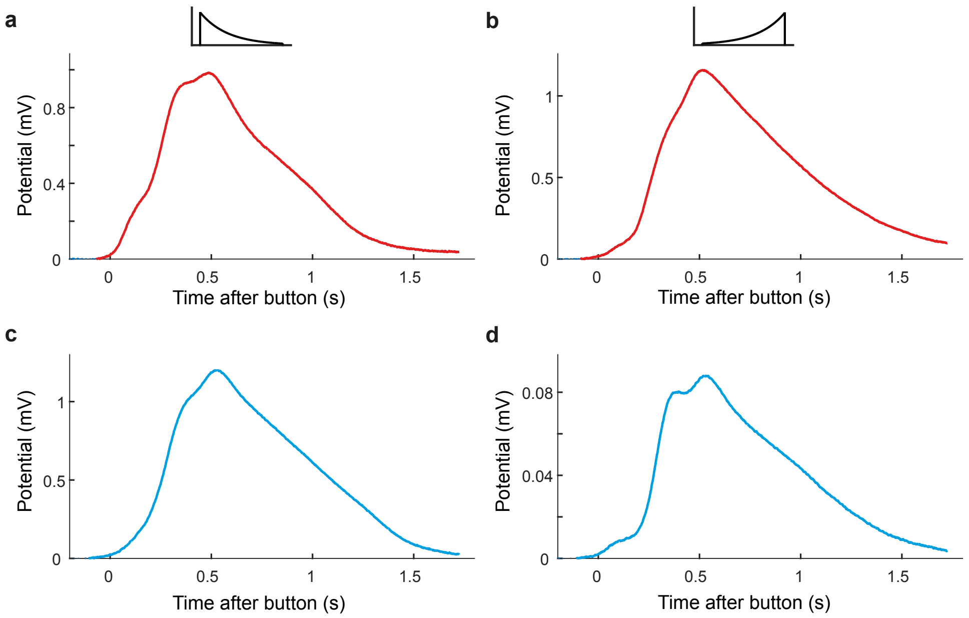

Supplementary Fig 4. | Eye-blinks distributed in time after the button press.
Time course of eye-blink signals measured with one electrode (channel EOGV) averaged across all trials and all participants. The eye-blink ERPs are distributed in time relative to the button press at t = 0 s. ERPs display maxima at ~0.5 s after the button press in **a)** auditory exponential condition (t_{max} = 0.485 s), **b)** auditory flipped exponential condition (t_{max} = 0.517 s), **c)** visual exponential condition (t_{max} = 0.522 s), **d)** visual flipped exponential condition (t_{max} = 0.525 s). Therefore, the correlation between alpha power and RT was not driven by eye blink activity. Source data are provided as a Source Data file.

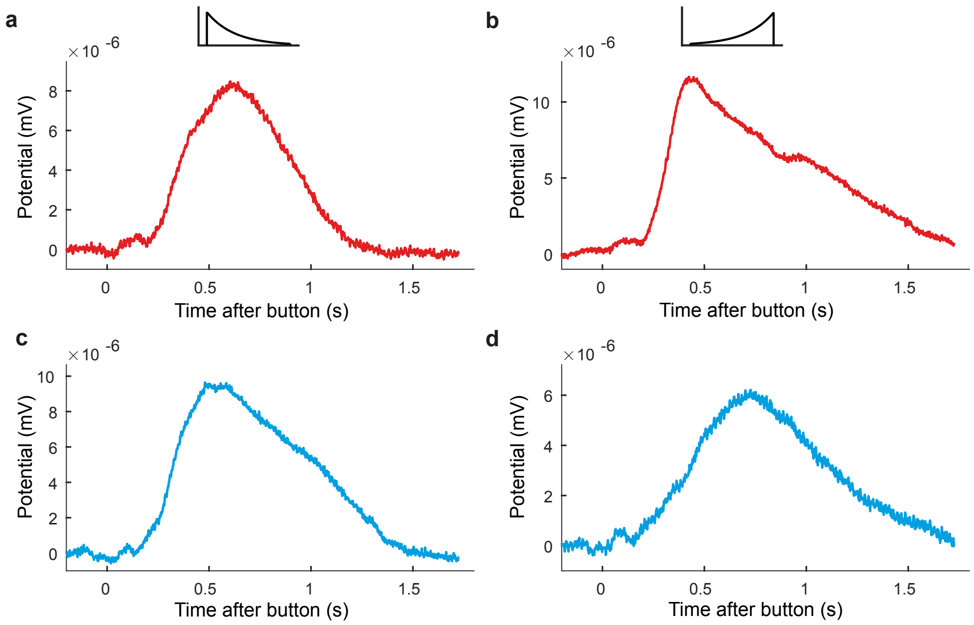

Supplementary Fig 5. | Eye-blinks distributed in time after the button press.

Time course of eye-blink signals measured with one electrode (channel EOGH) averaged across all trials and all participants. The eye-blink ERPs are distributed in time relative to the button press at t = 0 s. ERPs display maxima at ~0.5 s after the button press in **a)** auditory exponential (t_{max} = 0.605 s), **b)** auditory flipped exponential (t_{max} = 0.423 s), **c)** visual exponential (t_{max} = 0.480 s), and, **d)** visual flipped exponential conditions (t_{max} = 0.727 s). Therefore, the correlation between alpha power and RT was not driven by eye blink activity. Source data are provided as a Source Data file.

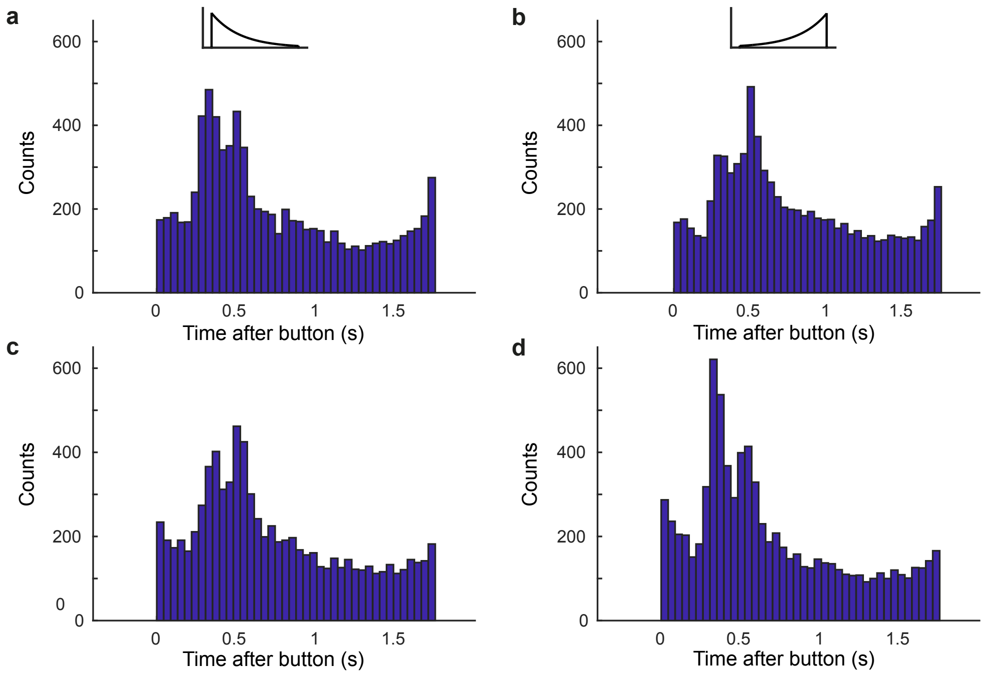

Supplementary Fig 6. | Eye-blinks distributed in time after the button press.

Histogram of time points at which eye-blink signal (electrode channel EOGH) is maximal, computed at the single-trial level over t = [0, 1.75] s. Eye-blinks are distributed in time relative to the button press at t = 0 s in **a)** auditory exponential, **b)** auditory flipped exponential, **c)** visual exponential, and, **d)** visual flipped exponential conditions. Therefore, the correlation between alpha power and RT was not driven by eye blink activity.

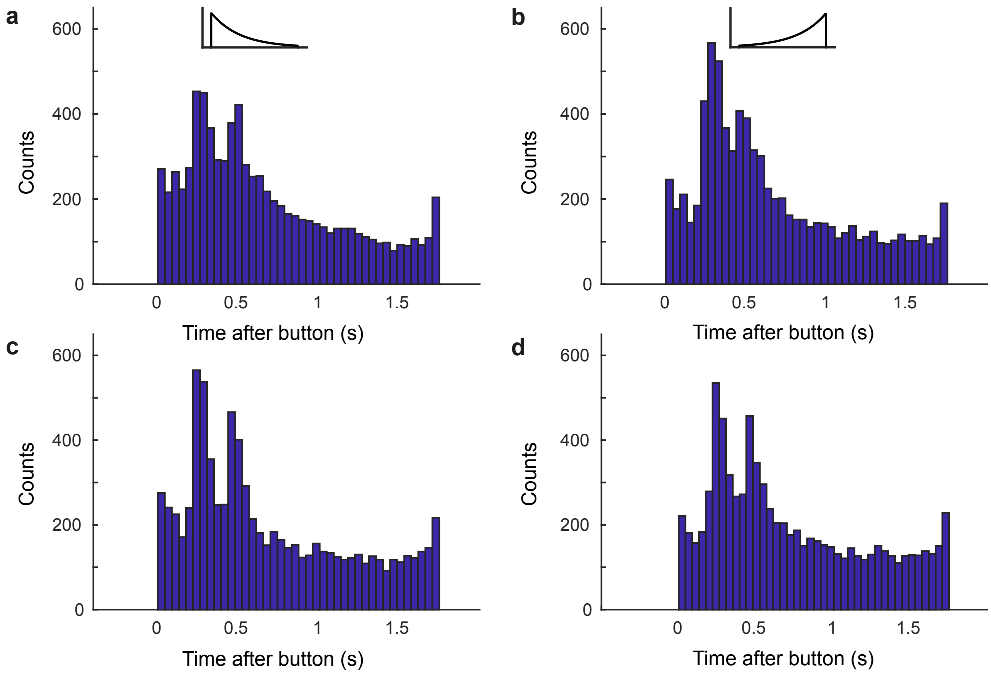

Supplementary Fig 7. | Eye-blinks distributed in time after the button press.

Histogram of time points at which pupil diameter is minimal (i.e. eyes closed), computed at the single-trial level over t = [0, 1.75] s. Time points of eye blinks are distributed in time relative to the button press at t = 0 s in **a)** auditory exponential, **b)** auditory flipped exponential, **c)** visual exponential, and, **d)** visual flipped exponential conditions. Therefore, the correlation between alpha power and RT was not driven by eye blink activity.

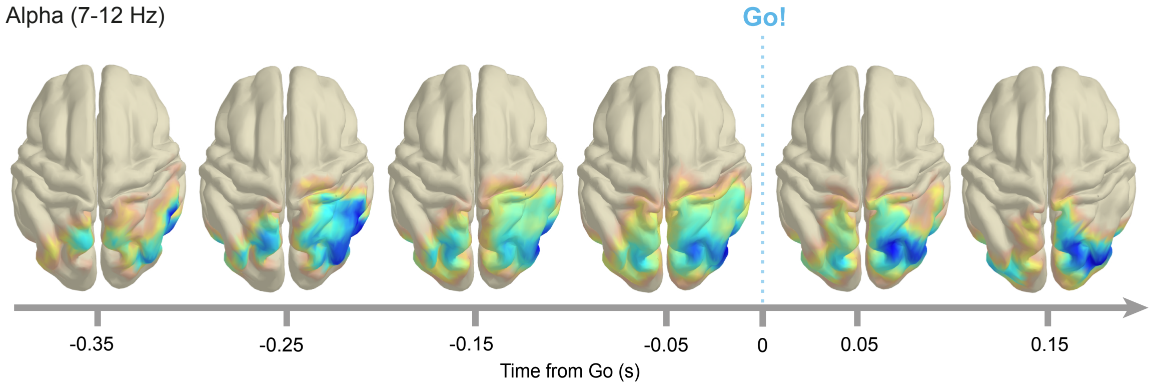

Supplementary Fig. 8 | Right lateralization of source-level correlation in the alpha band. Spearman correlation computed between single-trial RT and single-trial alpha power averaged across frequencies (7-12 Hz). Plots use an individual colormap for each time window (see Supplementary Fig. 9). For visualization, the average cortical surface of the 1200 Subjects Group Average Data[87] from the Human Connectome Project was used.

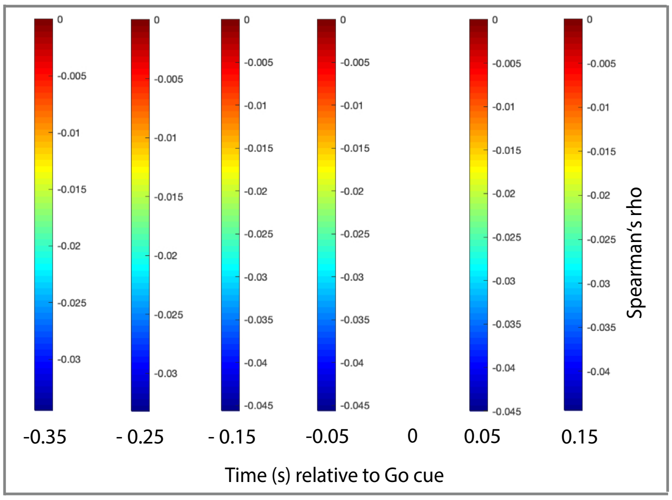

Supplementary Fig. 9 | Colorbars depict Spearman's rho over time. Individual colorbars for re-plotting of correlation (source-level alpha power and RT) shown in Fig. 6a (bottom row) and in Supplementary Fig. 8.

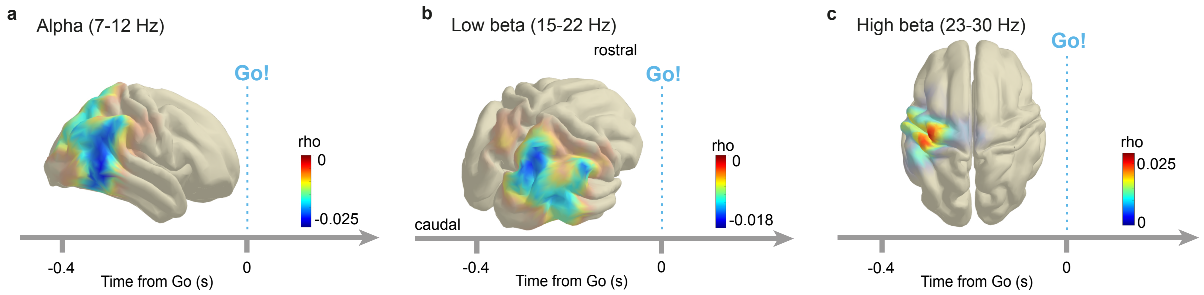

Supplementary Fig. 10 | Event PDF is represented in cortical key regions prior to the anticipated sensory cue. a. Cortical topography of across-condition Spearman correlation clusters (alpha (7–12 Hz) power, fit PDF-based model of RT), averaged across time (-0.4 to 0 s, P = [0.001, 0.006, 0.009, 0.01], Methods). Negative correlation covers inferior parietal lobule and the posterior middle temporal gyrus area. For visualization, the average cortical surface of the 1200 Subjects Group Average Data[87] from the Human Connectome Project was used. **b.** Across-condition Spearman correlation clusters (low beta (15–22 Hz) power, fit PDF-based model of RT), averaged across time (-0.4 to 0 s, P = [0.0035, 0.0085, 0.019, 0.021]). Negative correlation cluster in right superior parietal lobule (SPL). For visualization, the average cortical surface of the 1200 Subjects Group Average Data[87] from the Human Connectome Project was used. **c.** Across-condition Spearman correlation clusters (high beta (23–30 Hz) power, fit PDF-based model of RT), averaged across time (-0.4 to 0 s, P = [0.18, 0.012, 0.013, 0.001]). Positive correlation clusters in left sensorimotor cortex prior to Go cue. P values uncorrected. For visualization, the average cortical surface of the 1200 Subjects Group Average Data[87] from the Human Connectome Project was used.

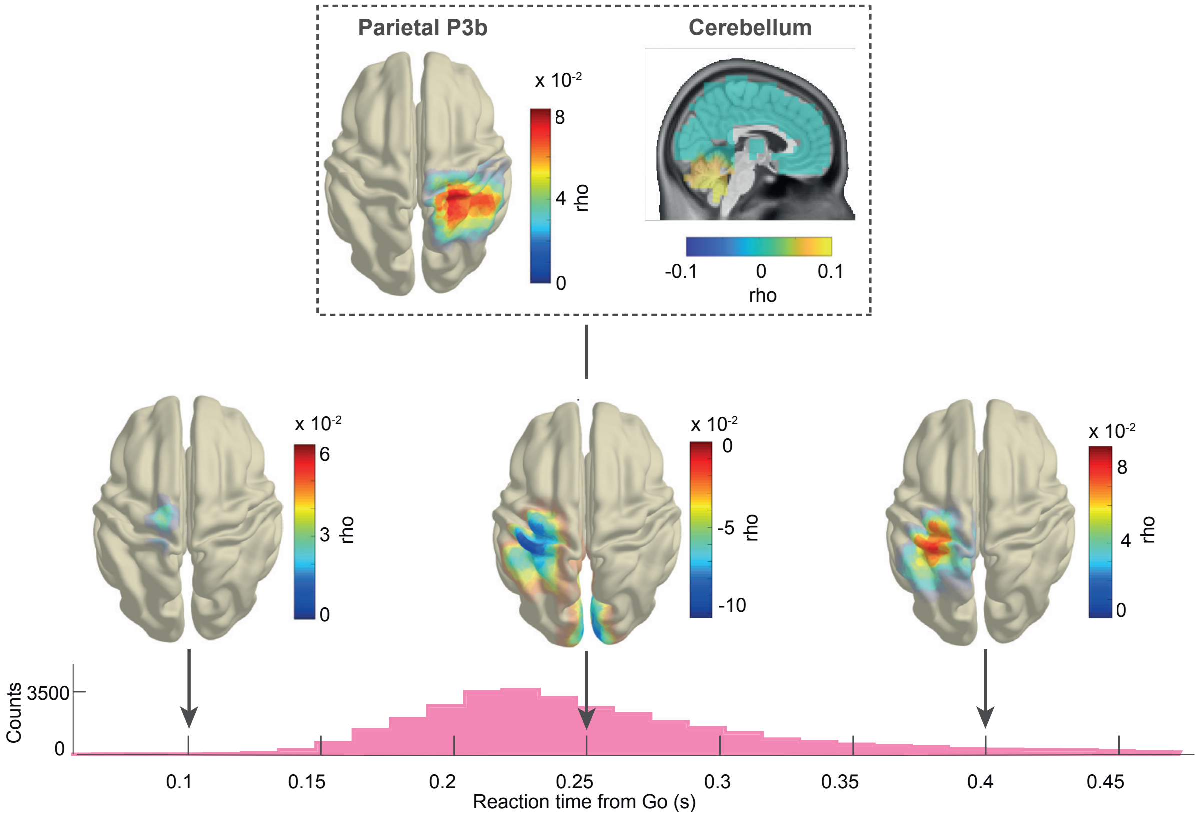

Supplementary Fig. 11 | Parietal, cerebellar and sensorimotor event-related fields correlate with fit PDF-based model of RT. Topography of across-condition source-level Spearman correlation clusters (ERF, PDF-based model of RT). At 0.25 s post-Go cue, positive correlation clusters in right parietal cortex (P3b, $P = 0.024$) and cerebellum ($P = 0.006$, both top) and a negative cluster in left sensorimotor cortex ($P = 0.002$, bottom). At 0.1 s, the cluster test identified a small number of sources in medial motor cortex but the cluster was not significant ($P = 0.70$). At 0.4 s, positive correlation clusters in left sensorimotor cortex ($P = 0.002$). Bottom: RT histogram from Fig. 2c for orientation. For visualization, the average cortical surface of the 1200 Subjects Group Average Data[87] from the Human Connectome Project was used.

Supplementary Methods

Source-level analysis of alpha and low beta power correlation with RT. To investigate the correlation between spectral power and RT at the sensor level, a cluster-based permutation test was performed across all channels, all frequencies and all time points. This analysis tested the null hypothesis that the correlation between RT and spectral power across all these dimensions is not significantly different from zero. The test rejected this null hypothesis, meaning that there is significant correlation somewhere within these dimensions. This mathematical space of significance is segmented according to predefined neighboring criteria in space, time and frequency. This segmentation identified two distinct clusters, one of positive and one of negative correlation (Fig. 5b top). By examining the correlation across frequencies, within the negative cluster, two prominent distinct negative peaks were identified (Fig. 5b bottom). One is in the alpha frequency range, the other in the lower beta frequency range which correspond to physiologically meaningful frequency bands. The positive cluster covers the high beta frequency range. These frequency bands were selected in order to investigate the location of this correlation in source space. This analysis was performed without any constraint on or prior selection of source space locations.

A note on correlation bias. In the ERF analysis, trials were aggregated in 30 frames of equal duration with respect to Go-time. The number of trials in each frame varied considerably, as the high probability frames contained many trials, whereas the frames with low event probability contained only few trials. Within each frame, the RT and the MEG data were respectively averaged for each subject, condition, channel and time point. So there is an obvious question whether averaging a different number of trials within each frame introduces a bias in the correlation between RT and MEG data.

We investigated this possible confound with simulated data resembling the distributions in the actual MEG data. The evoked MEG data had a distribution close to a Gaussian centered around zero while the TFR data resembled a highly skewed Weibul distribution with a long exponential tail. We simulated random data with similar distributions. RT was simulated by the 1/PDF linear model that was presented in the initial behavioral analysis.

The Go-time frames had the same distribution of trials as in the actual data. The simulated MEG data was then averaged within frames and the 30 frame values were correlated with the 30 RT simulated values. As the simulated MEG data was random, also the correlations with simulated RT values was expected to be around zero with no monotonic structure. Any monotonic

structure would hint towards an artifact from the averaging of a different number of trials per frame, as the trial number decreases or increases monotonically, depending on the event distribution (exponential: decreasing, flipped-exponential: increasing).

This procedure was repeated 10000 times with each time having new random simulated MEG data. The histogram of the resultant 10000 correlation values was plotted for the simulated TFR cases (Supplementary Fig. 11a) and ERF cases (Supplementary Fig. 11b) .

In the ERF case, no bias was observed in the distribution of rho, as it was centered round zero. In contrast, a significant bias was observed in the TFR case. In this case the distribution of rho was shifted away from zero towards negative values which should not be the case as the simulated MEG data was random. Clearly this is an artifact of averaging a different number of trials per frame. The bias seems to be dominant in the frames with the small number of trials, where the average tends to assume higher values, as compared to frames with many trials. The reason for this offset is the highly skewed distribution with some trials having very large values. Such trials have a dominating effect in frames with few trials and a much smaller effect in frames with many trials. That is why the bias seems to monotonically increase as one progresses from high probability frames to the less probable ones with fewer and fewer trials.

Based on these observations we decided in the actual data to follow different strategies for studying the correlation of the RT with the MEG ERF and the TFR data sets.

For the ERF data, as no bias seems to be present due to the averaging within frames, we proceeded and correlated the within-frames averaged, time-locked MEG data with the within-frames averages of RT.

For the TFR data, we correlated the data at the single-trial level, avoiding the monotonic bias in the average within bins.

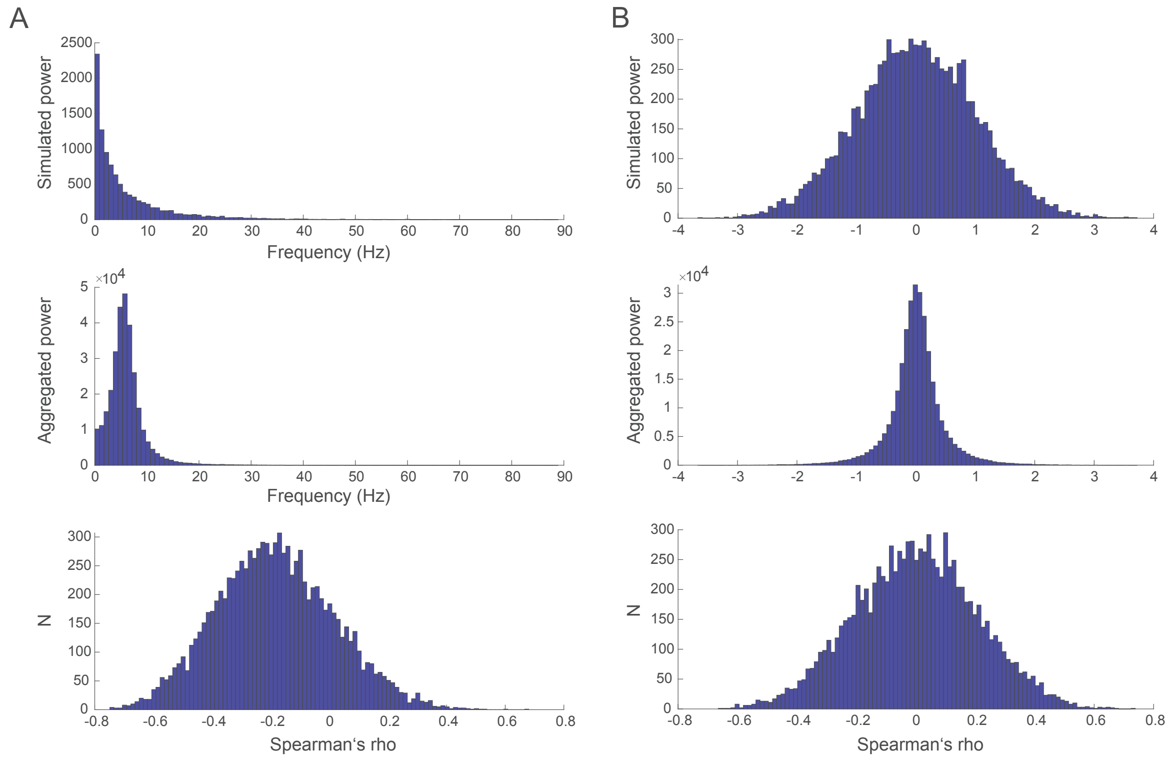

Supplementary Fig. 12 Data simulation. **a**) Simulated MEG power (arbitrary units) (top), aggregated within frames (arbitrary units) (middle) Spearman's rho computed on aggregated simulated power and 1/PDF (bottom). **b**) Simulated ERF data (arbitrary units) (top), aggregated within frames (arbitrary units) (middle), Spearman's rho computed on aggregated simulated ERF data and 1/PDF (bottom).
