# Supplementary material for: Neural signatures of temporal anticipation in human cortex represent event probability density
Source: Nat Commun. 2025 Mar 16;16:2602. doi: 10.1038/s41467-025-57813-7 (PMC11911442; doi:10.1038/s41467-025-57813-7)
Supplement: Supplementary file 2 — Description of Additional Supplementary Files [file 41467_2025_57813_MOESM2_ESM.pdf]

Description of Additional Supplementary Files

File name: Supplementary Data 1
Description: Reaction time data of the four experimental conditions.
