# Supplementary material for: Neural signatures of temporal anticipation in human cortex represent event probability density
Source: Nat Commun. 2025 Mar 16;16:2602. doi: 10.1038/s41467-025-57813-7 (PMC11911442; doi:10.1038/s41467-025-57813-7)
Supplement: Supplementary file 3 — Supplementary Data 1 [file 41467_2025_57813_MOESM3_ESM.zip › MeanRTMat_ReadMe.rtf]

MeanRTMat is a 3-dimensional matrix of dimensions 23 x 60 x 4. Dimension 1 is participants 1:23. Dimension 2 is reaction time averaged within each of the 60 different go times (0.4 to 1.4 s). Dimension 3 holds the 4 experimental conditions: 1: auditory exponential, 2: auditory flipped exponential, 3: visual exponential, 4: visual flipped exponential.There are N = 49 NaN (not a number) entries in MeanRTMat out of N = 5520 entries (0.89%). These NanNs indicate that in this combination of participant, go time, and condition, there was no reaction time that fulfilled the selection criteria (see Results). This reflects the probabilistic design: At the thin tails of the exponential and flipped exponential go time distributions, only a few trials of each go time were presented (down to N = 1). So if one of these single RTs did not meet the selection criteria, no RT at this specific go time entered analysis.
