# Supplementary material for: Neural signatures of temporal anticipation in human cortex represent event probability density
Source: Nat Commun. 2025 Mar 16;16:2602. doi: 10.1038/s41467-025-57813-7 (PMC11911442; doi:10.1038/s41467-025-57813-7)
Supplement: Supplementary file 4 — Reporting Summary [file 41467_2025_57813_MOESM4_ESM.pdf]

nature portfolio

Corresponding author(s): Matthias Grabenhorst

Last updated by author(s): 2025 2 5

Reporting Summary

Nature Portfolio wishes to improve the reproducibility of the work that we publish. This form provides structure for consistency and transparency in reporting. For further information on Nature Portfolio policies, see our Editorial Policies and the Editorial Policy Checklist.

Statistics

For all statistical analyses, confirm that the following items are present in the figure legend, table legend, main text, or Methods section.

n/a	Confirmed	
☐	☒	The exact sample size (*n*) for each experimental group/condition, given as a discrete number and unit of measurement
☐	☒	A statement on whether measurements were taken from distinct samples or whether the same sample was measured repeatedly
☐	☒	The statistical test(s) used AND whether they are one- or two-sided<br>*Only common tests should be described solely by name; describe more complex techniques in the Methods section.*
☒	☐	A description of all covariates tested
☐	☒	A description of any assumptions or corrections, such as tests of normality and adjustment for multiple comparisons
☐	☒	A full description of the statistical parameters including central tendency (e.g. means) or other basic estimates (e.g. regression coefficient) AND variation (e.g. standard deviation) or associated estimates of uncertainty (e.g. confidence intervals)
☐	☒	For null hypothesis testing, the test statistic (e.g. *F*, *t*, *r*) with confidence intervals, effect sizes, degrees of freedom and *P* value noted<br>*Give P values as exact values whenever suitable.*
☒	☐	For Bayesian analysis, information on the choice of priors and Markov chain Monte Carlo settings
☒	☐	For hierarchical and complex designs, identification of the appropriate level for tests and full reporting of outcomes
☒	☐	Estimates of effect sizes (e.g. Cohen's *d*, Pearson's *r*), indicating how they were calculated

Our web collection on statistics for biologists contains articles on many of the points above.

Software and code

Policy information about availability of computer code

Data collection	Data was collected using Psychophysics Toolbox Version 3, http://psychtoolbox.org/ running under MatLab version 2017b (The MathWorks, Natick MA, USA)
Data analysis	Data analysis was exclusively performed in MatLab (The MathWorks, Natick MA, USA), using versions 2019b and 2022b and the commercially available toolboxes (e.g. Statistics Toolbox) and the Fieldtrip toolbox (Robert Oostenveld, Pascal Fries, Eric Maris, and Jan-Mathijs Schoffelen. FieldTrip: Open Source Software for Advanced Analysis of MEG, EEG, and Invasive Electrophysiological Data. Computational Intelligence and Neuroscience, 2011; 2011:156869).

For manuscripts utilizing custom algorithms or software that are central to the research but not yet described in published literature, software must be made available to editors and reviewers. We strongly encourage code deposition in a community repository (e.g. GitHub). See the Nature Portfolio guidelines for submitting code & software for further information.

Data

Policy information about availability of data

All manuscripts must include a data availability statement. This statement should provide the following information, where applicable:

- Accession codes, unique identifiers, or web links for publicly available datasets
- A description of any restrictions on data availability
- For clinical datasets or third party data, please ensure that the statement adheres to our policy

The reaction time data used in the modeling are provided with this paper. The magnetoencephalography and and eye-tracking data are currently analysed in a dedicated project and will be made publicly available upon project completion. The specific data that support the findings of the paper are available from the corresponding author upon reasonable request.

Research involving human participants, their data, or biological material

Policy information about studies with human participants or human data. See also policy information about sex, gender (identity/presentation), and sexual orientation and race, ethnicity and racism.

Reporting on sex and gender	Participants' sex was determined based on self-reporting. No self-report on gender was performed. No sex- or gender-based analyses were performed as this was not part of the study design.
Reporting on race, ethnicity, or other socially relevant groupings	No socially-constructed categorization variables were used.
Population characteristics	24 healthy adults (15 female), aged 21-34 years, mean age 27 years, participated in the experiment. All were right-handed and had normal or corrected-to-normal vision, reported no hearing impairment and no history of neurological disorder.
Recruitment	Participants were recruited using a custom experiment-management software. The software was set to generate a group of right-handed participants whose age ranged between 18 and 35 years out of a pool of several thousand participants.
Ethics oversight	The experiments were approved by the Ethics Committee of the University Hospital Frankfurt.

Note that full information on the approval of the study protocol must also be provided in the manuscript.

Field-specific reporting

Please select the one below that is the best fit for your research. If you are not sure, read the appropriate sections before making your selection.

☒ Life sciences ☐ Behavioural & social sciences ☐ Ecological, evolutionary & environmental sciences

For a reference copy of the document with all sections, see nature.com/documents/nr-reporting-summary-flat.pdf

Life sciences study design

All studies must disclose on these points even when the disclosure is negative.

Sample size	The study aimed to quantify neural activity during temporal anticipation using magnetoencphalography. The sample size was determined based on previous work (Grabenhorst et al. Nat Commun, 2019, Grabenhorst et al. PNAS, 2021) and on the randomization procedure described below.
Data exclusions	One participant did not finish the experiment due to tiredness. This participant was removed from the analysis and a different participant was tested. One participant was excluded from the analysis because the participant's anatomical MRI data were corrupted and could not be retrieved.
Replication	The current experiment replicated our previous behavioral results (Grabenhorst et al. Nat Commun, 2019) which used a very similar experimental design.
Randomization	To control for order effects, the conditions (sensory modalities and probability distributions) were organized in a Latin square design, based on which modality and distribution were shuffled and balanced across participants.
Blinding	No blinding of the investigators during data acquisition and analysis was used.

Reporting for specific materials, systems and methods

We require information from authors about some types of materials, experimental systems and methods used in many studies. Here, indicate whether each material, system or method listed is relevant to your study. If you are not sure if a list item applies to your research, read the appropriate section before selecting a response.

Materials & experimental systems

n/a	Involved in the study
☒	☐ Antibodies
☒	☐ Eukaryotic cell lines
☒	☐ Palaeontology and archaeology
☒	☐ Animals and other organisms
☒	☐ Clinical data
☒	☐ Dual use research of concern
☒	☐ Plants

Methods

n/a	Involved in the study
☒	☐ ChIP-seq
☒	☐ Flow cytometry
☒	☐ MRI-based neuroimaging

Plants

Seed stocks	*Report on the source of all seed stocks or other plant material used. If applicable, state the seed stock centre and catalogue number. If plant specimens were collected from the field, describe the collection location, date and sampling procedures.*
Novel plant genotypes	*Describe the methods by which all novel plant genotypes were produced. This includes those generated by transgenic approaches, gene editing, chemical/radiation-based mutagenesis and hybridization. For transgenic lines, describe the transformation method, the number of independent lines analyzed and the generation upon which experiments were performed. For gene-edited lines, describe the editor used, the endogenous sequence targeted for editing, the targeting guide RNA sequence (if applicable) and how the editor was applied.*
Authentication	*Describe any authentication procedures for each seed stock used or novel genotype generated. Describe any experiments used to assess the effect of a mutation and, where applicable, how potential secondary effects (e.g. second site T-DNA insertions, mosiacism, off-target gene editing) were examined.*
